# Advances in Platelet Function Testing—Light Transmission Aggregometry and Beyond

**DOI:** 10.3390/jcm9082636

**Published:** 2020-08-13

**Authors:** Jessica Le Blanc, François Mullier, Caroline Vayne, Marie Lordkipanidzé

**Affiliations:** 1 Montreal Heart Institute Research Center, Montréal, QC H1T 1C8, Canada; jessica.le.blanc@umontreal.ca; 2 Faculty of Pharmacy, Université de Montréal, Montréal, QC H3C 3J7, Canada; 3 Université catholique de Louvain, CHU UCL Namur, Namur Thrombosis and Hemostasis Center (NTHC), Hematology Laboratory, 5530 Yvoir, Belgium; francois.mullier@uclouvain.be; 4 Department of Hemostasis, University Hospital of Tours, 37044 Tours, France; caroline.vayne@univ-tours.fr; 5 EA 7501 GICC, University of Tours, 37000 Tours, France

**Keywords:** platelets, platelet functional tests, light transmission aggregometry, standardization

## Abstract

Platelet function testing is essential for the diagnosis of hemostasis disorders. While there are many methods used to test platelet function for research purposes, standardization is often lacking, limiting their use in clinical practice. Light transmission aggregometry has been the gold standard for over 60 years, with inherent challenges of working with live dynamic cells in specialized laboratories with independent protocols. In recent years, standardization efforts have brought forward fully automated systems that could lead to more widespread use. Additionally, new technical approaches appear promising for the future of specialized hematology laboratories. This review presents developments in platelet function testing for clinical applications.

## 1. Introduction

Over the last century, platelet function testing has undergone several transformations. From the development of the Duke–Ivy bleeding time [1], to the invention of the light transmission aggregometer by Gus Born [2], to the newer high throughput advances in platelet function testing [3,4], capturing platelets in their natural ability to form aggregates in response to vascular injury remains a challenge. This article presents recent developments in platelet function testing, and while it is difficult to predict which approaches will translate into widespread clinical hematology laboratory use, aspects of standardization, automation, and point-of-care devices will be specifically highlighted.

## 2. International Guidelines for Light Transmission Aggregometry Standardization

The invention of the now gold-standard light transmission aggregometer has rapidly revolutionized platelet function testing. The seminal papers by Gus Born in the 1960s describe this invention in the simplest terms [2,5,6,7], and while the technology has seen improvements in bench footprint and user-friendliness over the years, the underlying methodology has remained largely unchanged. The standard in the identification and diagnosis of primary hemostatic defects, light transmission aggregometry (LTA) continues to be time-, labour- and blood sample-intensive, rendering its use limited to specialized hematology laboratories. Even so, international surveys have regularly highlighted a lack of standardization in laboratory practices, making the results difficult to extrapolate to other centers [8,9,10]. Indeed, the Platelet Physiology Scientific and Standardization Committee of the International Society on Thrombosis and Haemostasis has conducted the largest global survey on LTA practices [8], including 359 laboratories from 48 countries. In this survey, the methodology of blood collection, processing and analysis were observed in each center. The results of their report clearly demonstrate the need for methodological standardization among the different centers worldwide. In order to overcome this issue, the International Society on Thrombosis and Haemostasis has published an expert consensus for LTA standardization. This guidance includes several statements on pre-clinical variables to be considered and recommendation for blood collection, preparation of the platelet-rich plasma sample and the choice of platelet agonists for testing [10]. While this publication provides recommendations about technical procedures, Hayward et al. have developed a consensus guide on how to interpret LTA results [9]. Finally, the British Committee for Standards in Haematology has also published a guide about clinical investigation of heritable platelet function disorders [11]. Altogether, these three guidelines bring important standardization of the methodology and the interpretation of light transmission aggregometry testing, but in the absence of international standard reagents, or a quality assessment program for LTA, standardization between laboratories remains a challenge. Table 1 illustrates the differences in the recommended concentrations of activators and ristocetin for LTA in international guidelines [9,10,11,12,13,14].

## 3. Light Transmission Aggregometry Revisited

Light transmission aggregometry remains the gold standard platelet function test for clinical diagnosis of platelet function disorders. This technique determines platelet aggregation percentage in platelet-rich plasma by measuring the increase in light transmission in response to the addition of a platelet agonist to the platelet suspension. Recently, two developments have simplified the experimental procedure and are worth noting. First, the automation of light transmission platelet aggregation using dedicated software on routine laboratory instruments (such as the Sysmex CS-2x00 series, Norderstedt, Germany) introduces the possibility of running light transmission aggregometry without dedicated experienced personnel. The automated assay allows the user to select the agonists and their concentrations to be tested, in accordance with institutional, national, or international guidelines. The instrument then carries out automated light transmission aggregometry. In instances where it has been compared head-to-head with traditional light transmission aggregometry (Table 2), the Sysmex CS-2000i has shown repeatability, reproducibility and agreement with traditional aggregometers [15,16,17,18]. While the technology is certainly advantageous in terms of labour requirement, it still calls for an appreciable volume of platelet-rich plasma (140 µL per test) albeit less than traditional aggregometry (200–500 µL per test) [16]. Notwithstanding, the automated acquisition allows for concentration-response curves to be generated more readily than with traditional aggregometry. While the gain in personnel time is undeniable, the time to run the assays has remained sensibly the same, and the cost of reagents and consumables is higher than that of traditional aggregometry. Interpretation continues to rely on expert examination of aggregation tracings from a patient, in comparison with tracings from a healthy control, by an experienced hematologist or clinical pharmacologist. However, it is foreseeable that the automated assay could be run in non-specialist centers, with the results sent to a tertiary center for interpretation. The standardization of the automated assay could also alleviate some of the issues around reproducibility among laboratories worldwide.

The second development worth mentioning is that of high-throughput 96- to 384-well based platelet function assays, which allow a much broader overview of platelet function in significantly less time [3,4,19]. The plate-based assays offer the advantage of reduced platelet-rich plasma volume (50–100 µL per test for 96-well plates, 10 µL per test for 384-well plates) and time, as all assays are carried out simultaneously. Platelet function can be measured either kinetically or as an endpoint (e.g., after five minutes) and the results are converted into percentage of aggregation in a similar fashion to traditional light transmission aggregometry-based results. The large number of simultaneous aggregations that can be run on a plate make concentration–response curves to numerous agonists easy to generate, although these can be hard to interpret [20,21]. These assays remain restricted to research laboratories. Direct comparison of the 96-well plate assay with traditional light transmission aggregometry has revealed that the assays behave slightly differently in their sensitivity to agonists despite a similar methodological framework [22,23]. While these plate-based assays could be used as a preliminary screening assay outside of specialized centers, they should not be regarded as a replacement for traditional aggregometry.

## 4. Evaluation of Granule Defects

There is evidence that dense granule secretion defects can be misdiagnosed if relying solely on platelet aggregometry [25,26]. A modified version of light transmission aggregometry, the lumi-aggregometer, provides information on platelet secretion in parallel with platelet aggregation measures [25]. In this method, the ATP secreted by platelets is quantified using a luciferin/luciferase assay, while aggregation data is collected as in classical LTA. The combined analysis of platelet aggregation and secretion function by lumi-aggregometry enhances the detection of platelet disorders affecting dense granule release [27].

Several instruments are available to measure lumi-aggregometry (Chronolog series). However, there are few reports available in the literature on the validation and the performance of lumi-aggregometry. Lumi-aggregometry is affected by several variables including: concentration of luciferin/luciferase, concentration of agonists, volume of PPP and PRP, concentration of ATP standard, duration of incubation, duration of measurement and adjustment of platelet count of the PRP. As for LTA, non-parametric analyses are the preferred method to establish reference intervals for lumi-aggregometry [9,28,29]. Lumi-aggregometry estimates of platelet dense granule ATP release have a considerably higher CV (around 20–30%) than LTA. A recent report on 150 unique subjects who had multiple ATP release tests has shown that normal findings with all tested agonists were often confirmed by the second test, but impaired release with multiple agonists was confirmed in only some subjects. Inconsistent findings were thus common. The finding of impaired ATP release with two or more agonists on both tests was not associated with an increased likelihood of a definite bleeding disorder [30]. The variability in platelet dense granule ATP release findings amongst patients assessed for diagnostic purposes suggests that the test has limited value for diagnosing platelet disorders. However, these results should be confirmed by other groups before drawing firm conclusions.

In addition, caution should be used in patients with Quebec Platelet Disorder when assessing ATP secretion with the use of the Chronolume^®^ commercial reagent (containing 0.2 mg luciferin, 22,000 units d-luciferase plus magnesium sulphate, human serum albumin, stabilizers and buffer). Indeed, Hayward et al. found that addition of Chronolume^®^ consistently induced a secondary wave of aggregation in response to epinephrine in platelets obtained from patients with Quebec Platelet Disorder, whereas assessment of aggregation without Chronolume^®^ showed the expected absence of a secondary wave consistent with the pathology [31]. This finding however appeared to be restricted to patients with Quebec platelet disorder, as other investigators have reported no adverse effect of Chronolume^®^ addition in investigating platelet disorders [32,33]. These findings come from relatively small cohorts, and there remains an unmet need for systematic evaluation and standardization of methodologies for clinical laboratories using lumi-aggregometry.

A limitation of platelet lumi-aggregometry is that it does not distinguish between dense granule deficiency and primary secretion defects that may also rely on defects in signaling pathways. Therefore, assessment of the endogenous content of dense granules alongside lumi-aggregometry is important [11]. For that purpose, several strategies have been proposed, such as the measurement of platelet serotonin or nucleotides levels, using liquid chromatography, mass spectroscopy, immunoassays or flow cytometry. However, whole-mount transmission electron microscopy (TEM), which allows the direct quantification of platelet dense granules due to their calcium content, remains the gold standard and has been the subject of recent standardization efforts [34]. Despite being less accessible than LTA or ATP release assays in routine testing, this technology appears to be more sensitive and reproducible for detecting dense granule deficiencies associated with a bleeding tendency [35]. Beyond simply counting the number of dense granules per platelet, the measurement of their diameter could also be of importance in TEM as nearly 30% of bleeding patients with a normal number of dense granules may have smaller granules, leading to a reduced storage pool volume [36]. The study of platelet ultrastructure in TEM, although more complex to implement, is also very useful for the characterization of various platelet defects associated with the cytoskeleton and granule abnormalities, such as gray platelet syndrome, Paris-Trousseau syndrome, storage pool diseases, MYH9-related thrombocytopenia, or Wiskott–Aldrich syndrome, but is reserved to highly specialized laboratories [37].

Also of importance, while lumi-aggregometry assesses dense granule secretion, it fails to address secretion from α-granules, lacking a chromo-genic component [38]. In addition to TEM methods that allow assessment of α-granule number and morphology, immunofluorescence assays on blood smears are emerging as promising tools to characterize platelet α-granules [39]. Fluorescence microscopy on blood smears has been proposed to facilitate the diagnosis of several inherited platelet disorders associated with changes in platelet proteins, such as Glanzmann thrombasthenia, Bernard Soulier disease, and delta storage pool deficiencies, or macrothrombocytopenia associated with filamin A, GFI1B and β1-tubuline anomalies, for example [40]. Based on the preparation of standard peripheral blood smears followed by immunofluorescence labeling of various platelet components, this approach may be of interest in non-specialized centers worldwide (that may ship blood smears by regular mail to a specialized center), and particularly in pediatric population since it requires very low volumes of blood (<100 µL).

Genetic screening has also become an integral part of evaluating a patient presenting with inherited bleeding and platelet disorders [41,42], and has identified a number of important transcription factors involved in granule biogenesis and maturation that lead to bleeding disorders. Although informative, genetic screening cannot be taken in isolation, as the phenotype or functional readout of genetic findings is hard to predict. Characterization of α-granule contents by simple (ELISA) or multiple (e.g., Luminex^®^ or bead-based flow cytometry) immunological assays, can offer insights into the ability of platelets to secrete key vasoactive peptides in response to activation [27]. A frequently-used marker of α-granule fusion with the plasma membrane, flow cytometric assessment of P-selectin (CD62p) levels on platelets before and after activation with platelet agonists, may also indicate defects in α-granule biology. Although there is growing international expertise in assessing platelet α-granules, there are so far no universally accepted standardized assays that have been recommended in clinical practice [27].

## 5. Multiple Electrode Aggregometry

The impedance aggregometer has been described for the first time in 1980 [43]. In this technique, platelet aggregation is assessed by the change of electrical impedance in whole blood or in platelet-rich plasma, between two electrodes. Following agonist stimulation, platelets aggregate to the electrodes, impairing the conduction of electrical current between them. The development of a semi-automated system (Multiplate^®^, Roche Diagnostics) has allowed wide uptake of these instruments in hematology laboratories, especially for P2Y12 inhibitor monitoring [44]. Multiplate^®^ may be used to assess risk of bleeding or thrombosis during prolonged dual antiplatelet therapy and to shorten the time window to surgery following P2Y12 inhibitor discontinuation [45,46,47]. There is evidence, albeit limited, to support the use of impedance aggregometry for the diagnosis of severe platelet function disorders [48,49,50]. However, multiple electrode aggregometry was shown to be inferior to LTA for the detection and discrimination of mild platelet function disorders [49,51,52,53], since it provides no information about platelet shape change and the reversibility of aggregation. As such, its use is not recommended as a screening test for the diagnosis of bleeding disorders. Finally, several preanalytical and analytical variables affect the results provided by the instrument, including time-interval since blood drawing and analysis, type of anticoagulant, and platelet count [54,55].

## 6. Detection of Platelet Activation Markers by Flow Cytometry

Flow cytometry is another popular technique for platelet phenotyping. In contrast to aggregometry methods that study dynamic platelet aggregation, flow cytometry can shed insight on the platelet activation status, through analysis of the expression of activation markers. Two studies have compared light transmission aggregometry, with the measure of the expression of P-selectin and activated GPIIbIIIa on the platelet surface by flow cytometry in the detection of inherited platelet disorders [56,57]. The results highlight that flow cytometry has the advantage of requiring a smaller volume of blood and to not require platelet-rich plasma preparation. With a negative predictive value of 87%, flow cytometry analysis has the potential to be used as a screening test to perform before light transmission aggregometry. Thus, both studies have concluded that this technique provides complementary information for platelet function defects, even though further validation and standardization tests are required before use in diagnostic laboratories. In this respect, the setting of an appropriate threshold when studying platelet activation, the use of mean/median fluorescence intensity levels vs. % of positive platelets for a certain biomarker, the nature and concentration of agonists used to induce platelet activation, the use of fixatives (either before or after platelet staining) can all influence interpretation of flow cytometry-based platelet assessments and require standardization [58].

Another potential useful flow cytometry assay is the mepacrine assay which permits an evaluation of the incorporation and secretion capacities of platelets. The mepacrine captured by the δ-granules is then secreted upon stimulation of the cells with various agonists. Platelet fluorescence can thus be quantified by flow cytometry before and after stimulation. Mepacrine assays may be used to exclude platelet dense granule deficiency [59,60]. However, the mepacrine assay is also affected by a lack of standardization. Some of the variables include mepacrine concentration, temperature of mepacrine incubation, concentration and type of agonist used to stimulate platelets and mode of result expression. Alternatively, δ-granules secretion may be assessed by measuring the expression of CD63, a protein naturally present in the membrane of lysosomes and δ-granules, and which is translocated on the platelet surface upon platelet activation [61]. As well as lumi-aggregometry, this approach does not differentiate between storage pool deficiency and primary secretion defects, and its combination with mepacrine has been proposed to better characterize dense granules disorders [60]. Finally, flow cytometry is also used to detect the expression of phosphatidylserine on activated platelets in the case of a suspected diagnosis of Scott syndrome [62].

## 7. Microfluidics and Microscopy

**Figure 1:**
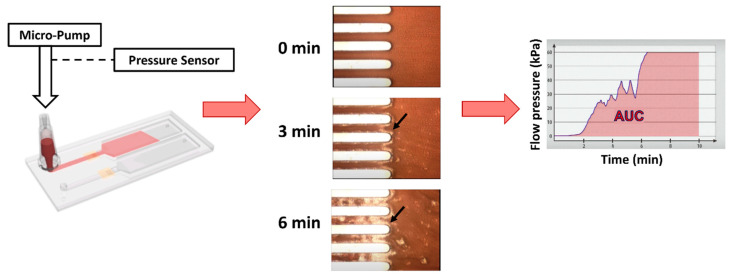
Principle of the T-TAS^®^ system for simplified evaluation of platelet plug formation in clinical laboratories. Reproduced with permission from Fujimori Kogyo, Co. Ltd, Tokyo, Japan.

Understanding platelet function as it occurs within a vessel requires elements of flow to be taken into account. Several point-of-care assays have been developed over the years that incorporate an element of shear, including the PFA-100/200^®^, Impact^®^—the cone and platelet analyzer, and Placor^®^ PRT. Of these, only the PFA-200^®^ remains clinically available, despite being fairly insensitive for the detection of mild platelet function defects [11,63]. In research laboratories, parallel-plate flow chambers have been in use since the 1970s and have allowed multiple discoveries to be made regarding the behavior of platelets under physiological and pathological flow [4]. It is only recently that microfluidic devices have been developed for clinical laboratory use. The principle of the assay is simple; it requires blood to be flowed over a surface coated with a thrombogenic substrate (usually collagen) and the assessment of platelet deposition and thrombus growth by microscopy. The Total Thrombus-formation Analysis System (T-TAS 01^®^) is one such example in clinical laboratory use. It is a flow-microchip chamber with thrombogenic surfaces that easily generates images for two-dimensional analysis of area covered by thrombi, in an imitation of a vessel wall injury [64,65,66,67,68,69]. Similar to the closure time reported by the PFA-200^®^, the T-TAS 01^®^ instrument reports the flow pressure waveform as the platelet plug obstructs blood flow through the microchip. Its dual-monitoring system adds real-time video imaging (Figure 1), which allows visual assessment of the thrombus formed under variable blood flow conditions. It has been successfully used for the diagnosis and characterization of von Willebrand disease [65], as a screening test for platelet storage pool disease [67,70], for monitoring of antiplatelet therapy [66,68,70], and for the prediction of periprocedural bleeding in patients undergoing coronary artery bypass surgery [71].

Several developments in the world of microfluidic devices are underway. A multi-modal approach with different coating proteins in addition to collagen appears promising to investigating platelet function under flow in clinical settings, including severe combined immune deficiency, Glanzmann thrombasthenia, Hermansky–Pudlak syndrome, MYH9-related disease, or grey platelet syndrome [72]. Microfluidic devices that incorporate endothelial cells could be useful in assessing platelet function in hematologic diseases such as sickle cell disease and hemolytic uremic syndrome [73]. These advances are yet to achieve sufficient standardization to reach the clinical laboratory, but they could dramatically change the way we assess platelet function in bleeding or thrombotic disorders.

## 8. Platelet Function Testing in Thrombocytopenia

It is not rare that low platelet counts hamper assessment of platelet function by traditional assays, as most platelet function tests are not reliable when platelet counts fall below the normal range [74]. Yet, previous research has shown that bleeding risk is not directly correlated with platelet count. For example, clinically significant bleeding occurred on 25% of days when platelet counts reached ≤ 5 × 10^9^/L, 17% of days when platelet counts varied between 6 and 80 × 10^9^/L, 13% of days when platelet counts ranged from 81 to 100 × 10^9^/L, and 8% of days with platelet counts ≥ 100 × 10^9^/L, in a study of patients with hematological or oncological disorders [75]. This suggests that other qualitative factors may contribute to the risk of bleeding in addition to low platelet counts in patients with thrombocytopenia.

Options for assessment of platelet function in patients with low and very low platelet counts rely on flow cytometric assays of platelet activation markers. In recent years, several flow cytometry approaches have been successfully used to assess platelet function in patients with severe chronic immune thrombocytopenia, showing that impaired platelet function is associated with bleeding, independent of platelet count [76,77,78]. However, it should also be noted that simply decreasing the platelet count induces an impaired platelet function phenotype as measured by flow cytometry, due to the loss of paracrine amplification of platelet responses by ADP release [79]. This indicates that flow cytometric assessment of platelet function is affected by platelet count, admittedly to a lesser extent than other traditional platelet function assays, and highlights the importance of deriving platelet-count-adjusted reference ranges.

Among the available platelet function assays, flow cytometry remains the superior approach to measure platelet function in thrombocytopenia, and several developments are underway to improve on the technology. One such example is the use of multiplex flow cytometry to assess the signaling pathways involved in platelet responses to multiple agonists [80]. Whether these high-throughput flow cytometry approaches will allow for the better characterization of platelet function in thrombocytopenia remains to be established. However, the technology is ready for implementation in larger cohorts, and possibly in patients with bleeding disorders.

## 9. Reference Ranges & Interpretation

An important part of a platelet function defect diagnosis remains establishing normal reference ranges for each test. Thus, the North American consensus guideline for medical laboratories performing LTA recommend to locally establish the normal range of the maximal aggregation percentage for each concentration of the activators, with a minimum of 40 healthy control volunteers [9]. While this recommendation compensates for technique variation between different medical centers, it is also noteworthy that several characteristics of the patient himself influence the normal reference range of platelet function testing results. Indeed, it is known that platelet count, structure and activity vary during aging [81,82], and that platelet function slightly differs between genders [82,83,84,85] and among different ethnic groups [85,86]. Other authors have also highlighted significant differences in aggregation curves in platelets of neonates and pregnant women compared to those of adults in the general population [87]. In an attempt to determine normal reference values in platelet function testing with flow cytometry, a study has found that the inter-individual variation is approximately 23% for P-selectin expression and 37% for αIIbβ3 activation [82], confirming the importance for the clinician to consider the characteristics of the patient for the interpretation of platelet function tests results. Finally, intra-individual day-to-day variation in platelet function are also observed, justifying the necessity to repeat at least once a test with an abnormal result to confirm the diagnosis.

## 10. Genetic Screening of Patients with Inherited Platelet Disorders

Genetic analyses are increasingly used in patients with bleeding disorders if there are strong clinical and biological arguments in favour of an inherited platelet function disorder [42]. The gene-target approach, which consists of studying a given gene, has long been used to confirm the diagnosis of various platelet function disorders associated with a typical biological and/or clinical phenotype, such a Glanzmann thrombasthenia or syndromic disorders for example. However, inherited platelet disorders are very heterogeneous, with more than 50 currently known genes [88], and in most cases, a single candidate gene cannot be found despite sometimes extensive family histories. In recent decades, next generation sequencing (NGS) has revolutionized the landscape of molecular diagnostics by increasing throughput, providing an unbiased genetic screen, and identifying rare variants not always accessible with other technologies [41]. Allowing much faster identification of known genetic defects, but also discovery of new defects, NGS is promising for overcoming diagnostic wandering [88]. However, it still suffers from limitations, such as lack of accessibility, high cost, and sometimes difficulties in assigning pathogenicity to novel identified variants, in addition to ethical debates around its use [41,89]. Notwithstanding, future laboratory assessment of individuals with inherited platelet function disorders will certainly rely on a mix of clinical, genetic, morphological, and functional investigations, altogether harnessing a more exhaustive platelet characterization.

## 11. Conclusions

There are still many challenges to accurately capturing in vivo platelet function with in vitro assays. Future assays will have to find a way to assess platelet interaction with the vessel wall, a likely contributor to certain bleeding phenotypes, independent of platelet aggregation profiles. The development of new platelet function assays is a high-risk endeavor, and not surprisingly, many assays that are promising in research settings do not make it into the clinical laboratory. Nevertheless, improvements in automation, standardization, and usability are likely to render platelet function testing available outside of specialized hematology laboratories in the next few years.

## Figures and Tables

**Table 1:** Recommended concentrations of activators and ristocetin for LTA in international guidelines.

	Final Concentration	Christie et al., 2008 [12]	Hayward et al., 2010 [9]	Harrison et al., 2011 [11]	Cattaneo et al., 2013 [10]	Alessi et al., 2017 [13]	Alessi et al., 2020 [14]
**ADP**	2 µM		X	X	X	X	X
	5 µM	X		X			
	10 µM		X			X	X
	100 µM					X	X
**Collagen**	1 µg/mL			X			
	2 µg/mL		X		X	X	X
	10 µg/mL					X	X
	25 µg/mL			X			
	Type:		Type I fibrillary	Type I	Horm	Not mentioned	Not mentioned
**Epinephrine**	5 µM	X	X	X	X	X	X
	10 µM		X		X		
	25 µM					X	X
**TRAPs**	10 µM			X	X	X	X
	50 µM					X	X
	Type:			PAR-1 (SFLLRN) 10–100 µM and PAR-4 (AYPGKF) 100–500 µM TRAPs	PAR1 (-AP)	TRAP-6	TRAP-6
**Arachidonic Acid**	1 mM	X (0.5–1.6)	X (0.5–1.6)	X (0.5–1.0)	X	X	X
**Thromboxane A2 analog U6619**	1 µM	X	X	X	X	X	X
	2 µM	X					
	3μM					X	X
	5 µM						X
	10 µM					X	X
**Ristocetin**	Low dose	≤0.6	0.5–0.6	0.5–0.7			
	High dose	0.8–1.5	1.2–1.5	1.2–1.5	1.2 *	1.2 *	1.2 *
**Collagen-related peptide (CRP)- Convulxin**	1 µg/mL						Concentration not provided
	2 µg/mL			X (0.01-1)			
**Gamma-thrombin**	50–200 ng/mL			X			
**Ca-ionophore A23187**	1.25–10 µmol/L			X			
**Phorbol 12-myristate 13-acetate (PMA)**	30 nmol/L			X			Concentration not provided

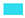
 Screening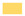 To diagnose complete P2Y12 deficiencies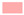 Can allow the TP receptor to be distinguished from TXA2 synthesis deficiencies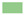 If arachidonic acid aggregation is abnormal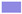 If γ thrombin is abnormal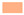 If abnormalities in the thrombin receptors, Receptors targets: Calcium mobilization and procoagulant function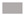 To check the correct functioning of the PKC pathway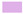 GPVI specific activator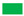 To diagnose CalDAG-GEFI deficiency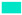 Thrombin receptors but without clotting
* If normal, then 0.5–0.7 mg/mL; if absent then 2 mg/mL

**Table 2:** Summary of studies comparing the automated light transmission aggregometers to traditional devices.

Study	Assessed Instrument	Reference Instrument	Samples	Agonists	Results	Comments on Assessed Instrument
LQ. Ling et al. [15]	Sysmex CS-2100i	Chrono-log Model 700 (Chrono-log Corporation, Havertown, PA, USA)	Pooled PRP from healthy subjects (n=8-10)	ADP 5 µM, AA 500 µg/mL, Col 2.5 µg/mL, Epi 5.4 µM, Risto 1.5 mg/mL (Hyphen Biomed)	Strong correlation between both instruments (Pearson’s r: 0.69 to 0.88) Good repeatability of CS-2100i (MA CV < 10%)	Inhibitory effect of PPP on aggregation induced by ADP, AA, Col, Epi Inhibitory effect of PS on ristocetin-induced aggregation Short turnaround time Low PC requirement in PRP: 80 × 10^9^/L
AS. Lawrie et al. [16]	Sysmex CS-2100i	AggRAM aggregometer (Helena Laboratories, Beaumont, TX, USA)	PRP from healthy subjects or patients on NSAID (n = 14) or clopidogrel (n = 2)	ADP 0.5–10 µM, AA 0.12–1.0 mM Col 0.5–10 μg/mL, Epi 0.5–10 µM, Risto 0.75–1.25 mg/mL (Hyphen Biomed)	Comparable dose-responses with each of the agonists with both instruments Comparable aggregation traces with samples from subjects under NSAID or clopidogrel Similar aggregation imprecision (MA and slope CV to ADP: 3–12%)	Influence of cuvette stirrer speed on the reaction sensitivity: optimum speed of 800 rpm No clinically significant changes in aggregation response for PC ranging from 150–480 × 10^9^/L in PRP, but poor sensitivity in case of PC <100 × 10^9^/L
C. Frère et al. [17]	Sysmex CS-2100i	APACT-4004 aggregometer (LABiTec, Ahrensburg, Germany)	PRP from patients suspected from PFD (n = 46) or with ACS (n = 62) receiving dual antiplatelet therapy	ADP 2.5–10 μM, AA 0.5 mg/mL, Col 3.3 μg/mL, Epi 10 μM, Risto1.25 mg/mL (Hyphen Biomed)	Significant correlations between both instruments (Pearson’s r: 0.38 to 0.98) Similar aggregation profiles with both systems in patients with bleedings (including 1 GT patient) Strong inter-agreement rates to detect low responders to thienopyridines or aspirin (weighted kappa> 0.70) Good intra-serial imprecision of CS-2000i (MA CV < 5% for each agonist)	Cuvette stirrer speed: 800 rpm
VE. Bret et al. [18]	Sysmex CS-2500	APACT-4004 aggregometer (LABiTec, Ahrensburg, Germany)	PRP from patients with suspected PFD, vWD or antiplatelet therapy (n = 49)	ADP 0.5–10 μM, AA 1 mM, Col 2 μg/mL, Risto 0.625 and 1.2 mg/mL (Hyphen Biomed)	Significant correlation between the two aggregometers (Passing and Bablok’s r: 0.48 to 0.90) More variable response using low concentrations of ADP (≤5 μM) with Sysmex CS-2500 Discrepancies with the low dose of ristocetin: excessive paradoxical agglutination with the Sysmex CS-2500 Good intra-serial imprecision of CS-2500 (MA CV to ADP: 1.5%)	Cuvette stirrer speed: 600 rpm
J. Stratmann et al. [24]	Sysmex CS-2100i	APACT-4004 aggregometer (LABiTec, Ahrensburg, Germany)	PRP from healthy subjects (n = 61) and from patients with known bleeding disorder (n = 20) or antiplatelet therapy (n = 42)	ADP 5 μM, AA 1 mM, Risto 1 mg/mL), Col 2 μg/mL, Epi 5 μM (Hyphen Biomed)	Significant MA correlation between both instruments with all subgroups and agonists tested (Pearson’s r ≥ 0.85) Weak or no correlation between both instruments in regard to lag time (Pearson’s r < 0.20) Systematic bias to lower measurements below a threshold of 50% MA with the CS-2100i Successful identification of patients with known bleeding disorder or antiplatelet therapy using the CS-2100i	Non-adjusted PRP Reading period of 600 s

AA: arachidonic acid, ACS: acute coronary syndrome, ADP: adenosine diphosphate, Col: collagen, CV: coefficient of variation, Epi: epinephrine, GT: Glanzmann thrombasthenia, MA: maximum aggregation, NSAID: nonsteroidal anti-inflammatory drugs (i.e., aspirin or ibuprofen), PC: platelet count, PFD: platelet function disorder, PPP: platelet poor plasma, PRP: platelet rich plasma, PS: physiological saline, Risto: ristocetin, vWD: von Willebrand Disease.
